# Acute development of cortical porosity and endosteal naïve bone formation from the daily but not weekly short-term administration of PTH in rabbit

**DOI:** 10.1371/journal.pone.0175329

**Published:** 2017-04-10

**Authors:** Hiroshi Yamane, Aya Takakura, Yukari Shimadzu, Toshiyuki Kodama, Ji-Won Lee, Yukihiro Isogai, Toshinori Ishizuya, Ryoko Takao-Kawabata, Tadahiro Iimura

**Affiliations:** 1 Laboratory for Pharmacology, Pharmaceutical Research Center, Asahi Kasei Pharma Corporation, Shizuoka, Japan; 2 Division of Analytical Bio-Medicine, Graduate School of Medicine, Ehime University, Shitukawa, Toon city, Ehime, Japan; 3 Laboratory for Safety Assessment and ADME, Pharmaceutical Research Center, Asahi Kasei Pharma Corporation, Shizuoka, Japan; 4 Division of Bio-Imaging, Proteo-Science Center (PROS), Ehime University, Shitukawa, Toon city, Ehime, Japan; 5 Medical Affairs Department, Pharmaceutical Business Administration Division, Asahi Kasei Pharma Corporation, Tokyo, Japan; 6 Division of Analytical Bio-Medicine, Advanced Research Support Center (ADRES), Ehime University, Shitukawa, Toon city, Ehime, Japan; 7 Artificial Joint Integrated Center, Ehime University Hospital, Shitukawa, Toon city, Ehime, Japan; University of California, Los Angeles, UNITED STATES

## Abstract

Teriparatide [human parathyroid hormone (1–34)], which exerts an anabolic effect on bone, is used for the treatment of osteoporosis in patients who are at a high risk for fracture. That the once-daily administration of teriparatide causes an increase in cortical porosity in animal models and clinical studies has been a matter of concern. However, it is not well documented that the frequency of administration and/or the total dose of teriparatide affect the cortical porosity. The present study developed 4 teriparatide regimens [20 μg/kg/day (D20), 40 μg/kg/day (D40), 140 μg/kg/week (W140) and 280 μg/kg/week (W280)] in the rabbit as a model animal with a well-developed Haversian system and osteons. The total weekly doses were equivalent in the low-dose groups (D20 and W140) and in the high-dose groups (D40 and W280). After the short-term (1 month) administration of TPDT, micro-CT, histomorphometry and three-dimensional second harmonic generation (3D-SHG) imaging to visualize the bone collagen demonstrated that daily regimens but not weekly regimens were associated with the significant development of cortical porosity and endosteal naïve bone formation by marrow fibrosis. We concomitantly monitored the pharmacokinetics of the plasma teriparatide levels as well as the temporal changes in markers of bone formation and resorption. The analyses in the present study suggested that the daily repeated administration of teriparatide causes more deleterious changes in the cortical microarchitecture than the less frequent administration of higher doses. The findings of the present study may have some implications for use of teriparatide in clinical treatment.

## Introduction

Teriparatide (TPTD) [human parathyroid hormone, hPTH (1–34)] exerts anabolic effects on bone when it is administered intermittently. It is used for the treatment of osteoporosis patients who are at a high risk for fracture [1–3]. The fundamental effects of PTH on the bone metabolism are the enhancement of bone turnover by the stimulation of both osteoblast-mediated bone formation and osteoclast-mediated resorption. It has been well established that the effects of the pharmacological actions of PTH on bone cells, and ultimately on the skeletal structure depend on the dosing regimen. The continuous infusion of PTH increases bone resorption over bone formation, thus causing the net loss of bone mass, while intermittent injection increases net bone mass in which bone formation is dominant over bone resorption [4, 5].

In Japan, TPTD can be administered once-daily or once-weekly for the treatment of osteoporosis. Both regimens have been reported to increase the bone mineral density (BMD) of the spine [6, 7] and reduce the risk of vertebral fractures [2, 3] and these effects appear to be comparable. However, the two regimens have different pharmacological effects on the markers of bone metabolism. The once-weekly administration of TPTD or hPTH_1-84_ leads to a relatively moderate increase in the levels of bone formation markers and a decrease in the levels of bone resorption markers, while the once-daily administration leads to marked increases in the markers of both bone formation and resorption [3, 6, 8, 9]. Thus, investigations into whether different TPTD dosing regimens have different effects on the bone microarchitecture and estimated bone strength have been warranted.

Our research group previously reported that the less frequent administration of TPTD (three-times-weekly) increased the BMD, trabecular number and thickness, and mechanical strength of the lumbar vertebra and femur in an ovariectomized (OVX) rat model [10]. This regimen also increased the levels of bone formation markers without an increase in the levels of bone resorption markers, which was in clear contrast with another study that reported that the once-daily hPTH_1-84_ increased both bone formation and resorption [11]. In a recent study in which TPTD was administered to young adult male mice in several distinct regimen settings, the frequency of TPTD administration was found to affect the structural pattern of bone formation. The high-frequency administration of TPTD was associated with the formation of thin trabeculae and porotic cortices, with a rapid increase in the BMD and accelerated bone remodeling. In contrast, the low-frequency administration of TPTD led to the formation of thicker trabeculae with the maintenance of thick cortices [12].

[7]and thinner cortices induced by increased intracortical remodeling as determinants of bone fragility, which is highly associated with the risk of non-vertebral fractures, while it is well-established that trabecular bone loss is associated with the risk of vertebral fractures [13–19]. Cortical porosity has been reported to develop through the once-daily administration of TPTD in clinical [7] and animal studies [20–23], which suggests that this regimen has a deleterious effect on the microarchitecture of cortical bone.

Bone marrow fibrosis is described as the expansion of a population of fibroblastoid cells on the endoseteal surface. It is observed in conditions of high bone turnover, such as Paget’s disease, fibrous dysplasia and renal osteodystrophy with secondary hyperparathyroidism [24–28]. The production of poorly mineralized extracellular matrix by these fibroblastoid cells onto the bone surface is thought to contribute to the impaired bone quality that is observed in these pathological conditions [27–29]. Experimentally, the continuous infusion of TPTD has been reported to lead to the development of obvious bone marrow fibrosis in rats [30] and the expression of the constitutively active form of parathyroid hormone/parathyroid hormone-related proteins receptors (PPR*TG) in transgenic mice [31].

It is not well documented, however, whether different frequencies of TPTD administration have different effects on the microarchitecture of the cortical bone, such as the development of cortical porosity and bone marrow fibrosis, which would be informative for managing the risk of non-vertebral fractures. To assess this issue, the most commonly used animal models (mice and rats) are limited because of their lack of Haversian systems and osteons. Thus, in this study, we used the rabbit, which is a bone remodeling animal with a relatively well-developed Haversian system and osteons as our animal model [20, 23]. We analyzed the short-term (1 -month) effects of the once-daily and once-weekly administration of TPTD at low and high doses in comparison to a vehicle control group to determine whether the deleterious effects on the cortical microarchitecture, such as the development of cortical porosity and intra-cortical marrow fibrosis, occur before the obvious increase in BMD that is expected in the later months of treatment. We concomitantly monitored the pharmacokinetics of serum TPTD levels as well as the temporal changes in the markers of bone formation and resorption.

## Materials and methods

### Animals and the preparation of bone specimens

Seventeen female New Zealand White rabbits (Kbl:NZW) were purchased from Kitayama Labs (Nagano, Japan) and were allowed to acclimatize with free access to water and food for 6 days before use. Throughout the experimental study, the animals were housed individually under a 12-h light/dark cycle with free access to water, but their food intake was restricted to 120g /day (LRC4, standard diet for rabbits; Oriental Yeast, Tokyo, Japan). The animals at 6 months of age were divided into 5 groups and were subcutaneously injected with 20 or 40 μg/kg of TPTD once-daily (D20 group [n = 3] and D40 group [n = 3], 140 or 280 μg/kg of TPTD once-weekly (W140 group [n = 3] and W280 group [n = 4]) for four weeks (Table 1). For the high-dose administration of TPTD, the dosage in once-daily treatment was set at 40 μg/kg/day based on a previous study [23], while the dosage of once-weekly TPTD treatment was set at 280 μg/kg/week; thus, the cumulative dose of TPTD each week was the same with the two treatment frequencies. In the W140 and W280 groups, saline was administered every day, except for the days on which TPTD was administered. Saline injections were administered to control animals (C group, n = 4) once daily and to each group as a vehicle.

**Table 1:** Dosing regimen settings of this study.

Group		Treatment	Dose	Frequency	Total dose per week	n
**1**	**C**	Vehicle	0 μg/kg	1/day	0 μg/kg	4
**2**	**D20**	TPTD	20 μg/kg	1/day	140 μg/kg	3
**3**	**D40**	TPTD	40 μg/kg	1/day	280 μg/kg	3
**4**	**W140**	TPTD	140 μg/kg	1/week	140 μg/kg	3
**5**	**W280**	TPTD	280 μg/kg	1/week	280 μg/kg	4

Six-month-old female rabbits were given 4 distinct subcutaneous dosing regimens of TPTD (D20: 20 μg/kg/day, n = 3, D40: 40 μg/kg/day, n = 3, W140: 140 μg/kg/week, n = 3, W280: 280 μg/kg/week, n = 4), Daily saline injection was given to a control group (C: 0 μg/kg/day, n = 3).

Calcein (Dojindo Laboratories, Kumamoto, Japan) was subcutaneously injected into each animal at a dose of 10 mg/kg body weight on days 13 and 5 before sacrifice for bone double labeling (1-7-1-3). After the dosing period, the animals were euthanized by exsanguination under anesthesia using thiopental. The left and right tibiae were collected for a micro-CT analysis and histomorphometry, respectively. The kidneys, thyroid and parathyroid of all animals were collected and fixed in 10% neutral buffered formalin for a histopathological examination. The left tibiae were packed in plastic bags and stored at -30°C until use in a micro-CT analysis and mechanical testing. The right tibiae were fixed in 70% ethanol, stained with Villanueva bone stain, dehydrated in a graded ethanol series, defatted in acetone, and embedded in polymethyl methacrylate (Wako Pure Chemical Industries, Osaka, Japan). Thin ground sections of 10–20 μm in thickness were prepared using a micro cutting machine and a grinding machine (EXAKT, Germany) from a cross-section at a plane that was 3 mm proximal from the tibiofibular junction, and were subjected to bone histomorphometry. Right iliac crest were also fixed in 70% ethanol and processed for Vilanueva bone stain, dehydration, defattation, polymethyl methacrylate embedding and sectioning to 5 μm for three-dimensional second harmonic generation (3D-SHG) imaging.

The experimental protocols were approved by the experimental animal ethics committee at Asahi Kasei Pharma Corporation and were conducted in accordance with the guidelines for the management and handling of experimental animals.

### Histopathological examination, and biochemical analysis for renal function

The fixed specimens that were used for the histopathological examinations were embedded in paraffin, sectioned, stained with hematoxylin and eosin, and examined under light microscopy using the standard procedures.

The serum samples collected for measurement of bone metabolic markers were also subjected to biochemical analysis to examine renal function. Serum blood urea nitrogen (BUN) and creatinine were measured using an autoanalyzer (Hitachi 7180 chemistry analyzer; Hitachi Ltd, Tokyo, Japan).

### Blood and urine sampling

Blood samples were collected from auricular veins before dosing (0) and at 5, 15, 30 minutes, 1, 2, 6 and 24 hours after the initial administration of TPTD on day 1 (in all groups); and on days 3 and 7 after the initial administration of TPTD (in the DV, W140 and W280 groups) on the 1^st^ day. The samples were centrifuged at 1600 x g, at 4°C for 10 minutes and plasma samples were collected in order to measure the concentration of TPTD. The samples were centrifuged at 1600 x g, at 4°C for 10 minutes and plasma samples were collected in order to measure the concentration of TPTD.

Blood samples in all groups were collected at 0, 6, 24 hours after the administration of TPTD on days 1 and 22. Blood samples in the C, W140 and W280 groups were also collected at days 3 and 7 after on the 1^st^ and 22^nd^ days of administration. The samples were centrifuged at 1700 x g, at 4°C for 15 minutes in tubes with clot activator and serum was collected in order to measure the concentration of osteocalcin.

In all groups, the animals were placed in metabolic cages and urine samples were collected for 12 hours from 0 to 12 hours and from 12 to 24 hours after the administration of TPTD on days 0 (before dosing), 1 and 29 of administration. We failed to collect urine samples from 1 of the 4 animals in the C group, 2 of the 3 animals in the D20 group and 1 of the 3 animals in the D40 group from 0 to 12 hours after the administration of TPTD on day 29. The urine samples were centrifuged at 400 x g, at 4°C for 5 minutes and the supernatant was collected in order to measure the concentration of DPD.

### The measurement of the plasma TPTD concentration

Teriparatide acetate was synthesized at Asahi Kasei Pharma Corporation (Tokyo, Japan) and was used as a standard substance. The teriparatide acetate concentration in the rabbit plasma was quantified using a Rat PTH IRMA Kit (Immutopics, Inc., San Clemente, CA) according to the manufacturer’s instructions. The pharmacokinetic parameters were calculated using a non-compartmental model (Phoenix WinNonlin; Pharsight, Mountain View, CA).

### The measurement of markers of bone metabolism

The serum level of osteocalcin (OC), a bone formation marker, was measured using a Gla-osteocalcin ELISA system (Takara Bio, Tokyo, Japan). The urinary level of deoxypyridinoline (DPD) was measured using Osteolinks DPD (DS Pharma Biomedical) and was normalized to the creatinine concentration. The assays were performed according to the manufacturers’ instructions. Concentrations of urine DPD and serum OC were demonstrated as percentage of pre-dosing (on day 0) values.

### Micro-Computed Tomography (CT)

A cone-beam X-ray micro-CT system (ScanXmate-RB090SS150; Comscantecno, Kanagawa, Japan) was used to obtain CT images of the left tibiae using the following settings: tube voltage, 70 kV; tube current, 100 mA; and resolution, 19.1 μm/voxel. Three-dimensional images were reconstructed and analyzed using the TRI/3D-BON software program (RATOC System Engineering, Tokyo, Japan). We analyzed regions that were 3 mm proximal from the tibiofibular junctions (height, 1 mm) perpendicular to the axes of the tibial shafts. The cortical bone structure was determined in the samples and the following parameters were measured: total tissue volume (Tv, mm^3^), cortical bone volume (Cv, mm^3^), cortical bone ratio (Cv /Tv, %), cortical bone thickness (Ct.Th, μm) and cortical porosity (Ct.Po, %), periosteal perimeter (Ps.Pm), endosteal perimeter (Es.Pm) and the moment of inertia.

### Histomorphometry

The following parameters of the right tibiae were measured using an image analysis system (Histometry RT Camera; System Supply, Nagano, Japan) and ImageJ (NIH, Bethesda, MD. USA): the periosteal single-labeled perimeter (Ps.sL.Pm), the periosteal double-labeled perimeter (Ps.dL.Pm), the periosteal eroded perimeter (Ps.E.Pm), the periosteal quiescent perimeter (Ps.Q.Pm), the endosteal single-labeled perimeter (Es.sL.Pm), the endosteal double-labeled perimeter (Es.dL.Pm), the endosteal eroded perimeter (Es.E.Pm), the endosteal quiescent perimeter (Es.Q.Pm), the void-labeled perimeter (Vd.L.Pm) and the void eroded perimeter (Vd.E.Pm). The value of Vd.L.Pm and Vd.E.Pm were divided by that of the cortical bone area (B.Ar) of the region of interest. In addition, the periosteal and endosteal perimeters with an unusual calcein labeling pattern, showed a complicated structure that was different from both the single- and double-labeled perimeter and which was frequently observed in the once-daily treated groups. These were measured as the “periosteal multiple labeled perimeter (Ps.mL.Pm)” and “endosteal multiple labeled perimeter (Es.mL.Pm)”, respectively. The histomorphometric measurements for the periosteal and endosteal regions were obtained from the entire cortical surface. The measurements for the intracortical void regions were obtained from the anteromedial one-eighth of the cortical area, where the increase in porosity induced by TPTD was the most evident.

### Bright field imaging with Differential Interference Contrast microscopy (DIC) and deconvolution fluorescence imaging

Calcein-labeled undecalcified bone sections were subjected to imaging acquisition using a microscopy system, ECLIPSE Ni (Nikon, Tokyo, Japan) equipped with differential interference contrast microscopy (DIC) and objectives (Nikon), as follows: Plan Apo λ 10x (numerical aperture [N.A.] = 0.45), Plan Apo λ 20x (N.A. = 0.75), and Plan Apo λ 40x (N.A. = 0.95). The fluorescence signals were obtained using filter sets, GFP-B (excitation: 460–500 nm, DM: 505 nm, emission: 510–560 nm; Nikon) for calcein, and TxRed (excitation: 540–580 nm, DM: 595 nm, emission: 600–660 nm; Nikon) for auto-fluorescence derived from soft tissue. Tiling fluorescence imaging to acquire the entire, high-contrast view of the tissue sections was carried out using a Plan Apo λ 10x objective (N.A. = 0.45). The frame size of a single scan was 1280 × 1024 pixels with an 8-bit color depth. The fluorescence and DIC images were sequentially acquired, with a pixel size of 0.64 μm. A total of 4 × 7 images were combined for tiling to obtain large images of whole tissue sections at a high resolution. Image processing, including deconvolution, was performed using the NIS-elements AR (Nikon) imaging software program.

### Three-Dimensional Second Harmonic Generation (3D-SHG) imaging by multi-photon microscopy

An upright multi-photon excited microscopy system (A1RMP, Nikon Corporation, Japan) situated at the Imaging core facility of The Institute of Medical Science, The University of Tokyo was employed to acquire bone collagen-derived SHG signals and two-photon excited auto-fluorescence signals from Calcein-labeled undecalcified bone sections. The microscope was equipped with a water-immersion objective lens (Apo LWD 40x, numerical aperture: 1.15 WIλS, Nikon) and a Chameleon laser oscillator (COHERENT, Inc. CA). The excitation wavelength was 880 nm. The detection wavelengths were 420–425 nm and 500–550 nm for the acquisition of collagen-derived SHG signals and auto-fluorescence signals from soft tissue, respectively. Three-dimensional (3D) images were acquired by taking 20 optical slices with a step size of 1000 nm. Image processing including 3D projection, 3D rendering and Deconvolution was performed using the NIS-elements AR (Nikon) imaging software program.

### The measurement of the bone mineral density

Bone mineral densities (BMD) of the collected left tibia, left femora and lumber vertebrae (LV) 4-LV6 were monitored using dual-energy X-ray absorptiometry (DXA) (DCS-600EX-IIIR, Aloka, Tokyo, Japan). The whole samples were scanned at a pitch of 2 mm. The BMD (mg/cm^2^) was then calculated from the bone mineral content (mg) and bone area (cm^2^).

### Bone mechanical properties

The fibulae were cut off from the left tibiae by a micromotor (Volvere GX NE22, Nakanishi, Japan). After the preprocessing, the samples were subjected to three-point bending test. The samples were set on supports 32 mm apart which were attached to a testing machine (AUTOGRAPH AGS-5kNX, Shimadzu, Japan) such that dorsal aspects were upward, and load was applied on points 3 mm proximal from tibiofibular junctions at a constant speed of 10 mm/min. The load and displacement curve were recorded and the following parameters were calculated using TRAPEZIUM LITE EX software (Shimadzu): maximum load (N), stiffness (N/mm), and Energy absorption (Energy, mJ). Energy absorption was defined as Energy absorbed until load reached for the recorded maximum values.

### Statistical analysis

The data from the bone metabolic marker measurements and the micro-CT analysis were expressed as the mean ± SD. The time-course data of serum osteocalcin in all groups at 0–24 hours after the administration of TPTD on days 1 and 22, and the time-course data in the C, W140 and W280 groups at 3–7 days after the administration of TPTD on days 1 and 22, were together analyzed using a two-way ANOVA comparing the differences among groups and time points. The TPTD-treated groups were compared with the DV group using Dunnett’s test at each time point after the confirmation of a significant difference by two-way ANOVA. For all groups, the urine DPD data from 0 to 12 hours and from 12 to 24 hours on days 1 and 29 were analyzed using a two-way ANOVA, and the differences between each group and the C group were then analyzed using Dunnett’s test at each time point after the confirmation of a significant difference by two-way ANOVA. Because we could only obtain a urine sample from 1 of the 3 animals in the D20 group from 0 to 12 hours after the administration of TPTD on day 29, we excluded D20 group from the statistical analysis and compared the D40, W140 and W280 groups with the C group at this time point. The micro-CT parameters were analyzed using a one-way ANOVA, and the TPTD-treated groups were then compared to the C group using Dunnett’s test. All of the statistical analyses were performed using the GraphPad Prism software program (version 6.05 for Windows; GraphPad Software, San Diego, CA, USA). *P* values of < 0.05 were considered to indicate statistical significance.

## Results

### The pharmacokinetic profiles of the different TPTD dosing regimens

**Fig 1:**
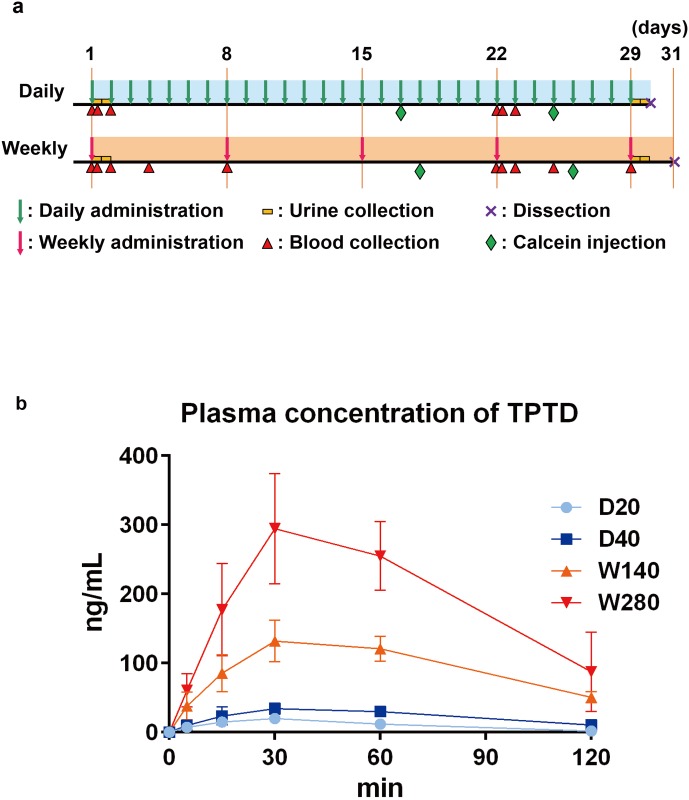
The time schedule of the experiments and sampling, and the pharmacokinetic profiles of the four distinct dosing regimens. (a) The time schedule of the administration of TPTD, calcein labelling and urine and blood sampling are shown. (b) Six-month-old female rabbits were subjected to one of four TPTD dosing regimens. TPTD (20 μg/kg/day once-daily, 40 μg/kg/day once-daily, 140 μg/kg/week once-weekly or 280 μg/kg/week once-weekly) was administered subcutaneously and the time-course changes in the plasma levels of TPTD were monitored. The data are shown as the mean ± SD (n = 3–4).

The plasma concentrations and pharmacokinetic parameters of TPTD during the administration of TPTD under four distinct regimens (D20, D40, W140 and W280) are shown in Fig 1b and Table 2 (and S1 Table). The plasma levels of TPTD rapidly increased after the administration of TPTD in the daily regimens (D20 and D40); to reach a maximum concentration (Cmax) of 19.78±5.67 and 34.30±8.19 ng/mL, at 30 min post-dose (Tmax), respectively. Under the weekly regimens, the Cmax values of the W140 and W280 groups were 133.27±27.96 and 314.59±67.85 ng/mL, respectively, which were 6.5 to 9.2 times higher in comparison to the equivalent weekly total dosage regimens (D20 and D40).

**Table 2:** Pharmacokinetic parameters of plasma TPTD.

Parameters	Units	20 μg/kg	40 μg/kg	140 μg/kg	280 μg/kg
**T**_**1/2**_	**hour**	**0.44 ± 0.15**	**0.93 ± 0.32**	**0.93 ± 0.16**	**0.69 ± 0.27**
**T**_**max**_	**hour**	**0.50 ± 0.00**	**0.50 ± 0.00**	**0.67 ± 0.29**	**0.75 ± 0.29**
**C**_**max**_	**ng/mL**	**19.78 ± 5.67**	**34.30 ± 8.19**	**133.27 ± 27.96**	**314.59 ± 67.85**
**AUC**	hour*ng/mL	**21.12 ± 3.67**	**46.75 ± 10.74**	**187.89 ± 35.46**	**389.67 ± 75.61**

T_2/1_: Elimination half-life, T_max_: Estimated maximum drug concentration time, AUC: Area under the concentration-time curve.

Furthermore, estimated Tmax values of the W140 and W280 group (40 and 45 min post-dose, respectively) was longer. The plasma concentrations of TPTD under the four distinct regimens (D20, D40, W140 and W280) then declined with a half-life (T1/2) of 0.44±0.15, 0.93±0.32, 0.93±0.16 and 0.69±0.27 h post-dose, respectively. Under all of these regimens, the TPTD concentrations, after reaching Cmax, exponentially declined to less than 40% of Cmax by 2h.

### No obvious toxic effects by any regimens of TPTD administration by histopathological and biochemical examinations

Because Cmax and AUC under weekly dosing regimens (W140 and W280) were extremely high in comparison to those observed in clinical use and previous rabbit studies [20, 23], it is possible that the administration of TPTD had some toxic rather than pharmacological effects. Thus, we carried out general histopathological examinations on all of the rabbits specimens that were used in our current study (Table 3). No obvious toxic effects were observed in any specimens.

**Table 3:** Histopathological examination.

Organ	Findings	Vehicle control 0 μg/kg				Once-daily TPTD 20 μg/kg			Once-daily TPTD 40 μg/kg			Once-weekly TPTD 140 μg/kg			Once-weekly TPTD 280 μg/kg			
		1	2	3	4	1	2	3	1	2	3	1	2	3	1	2	3	4
**Kidney**	**Degeneration/regeneration, tubule**	**-**	**-**	**-**	**-**	**-**	**-**	**-**	**-**	**-**	**-**	**-**	**-**	**-**	**-**	**-**	**-**	**-**
	**Dilatation, distal/collecting tubule**	**-**	**-**	**-**	**-**	**-**	**-**	**-**	**-**	**-**	**-**	**-**	**-**	**-**	**-**	**-**	**-**	**-**
**Thyroid**	**Pathological change**	**-**	**-**	**-**	**-**	**-**	**-**	**-**	**-**	**-**	**-**	**-**	**-**	**-**	**-**	**-**	**-**	**-**
**Parathyroid**	**Ectopic tissue, thymus**	**P**	**-**	**-**	**-**	**-**	**-**	**-**	**-**	**-**	**-**	**-**	**-**	**-**	**-**	**-**	**-**	**-**

Kidney, thyroid and parathyroid were collected from all animals and subjected for histopathological examination.

We also carried out blood chemical analysis to examine renal function. The level of serum blood urea nitrogen (BUN) and serum creatinine were not significantly affected by TPTD in any regimen group (S2 Table), suggesting that no obvious toxic effect of TPTD on renal function was occurred in our dosing regimens in this study.

### The marked response of the markers of bone metabolism to daily regimens at later time points

**Fig 2:**
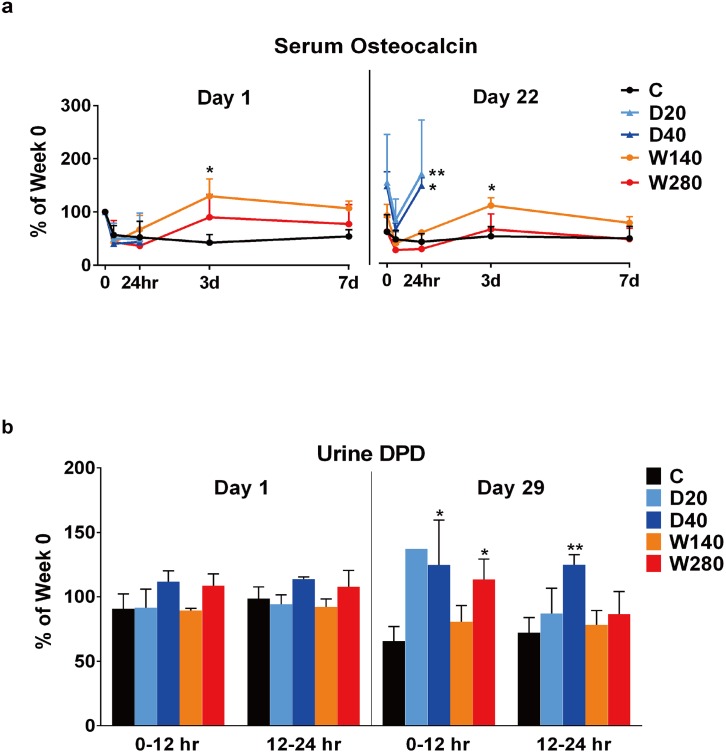
Temporal changes in the markers of bone metabolism. (a) The temporal changes in the serum levels of osteocalcin (OC) after the administration of TPTD on days 1 and 22 are shown. In the daily regimen groups (D20 and D40), the OC levels at 0 (pre-dosing), 6 and 24 h after the administration of TPTD were plotted. In the weekly regimen groups (W140 and W280) and the vehicle control (C) group, the OC levels at 0 (pre-dosing), 6 and 24 h, and 3 and 7 days after the administration of TPTD were plotted. (b) The changes in the urinary deoxypyridinoline (DPD) level after the administration of TPTD on days 1 and 29 are shown. The urine samples were collected for 12 hours, from 0 to 12 hours and from 12 to 24 hours after the administration on each day, and the rates of change in the urine DPD levels in comparison to pre-dosing (before the initial administration) were determined. (a) and (b): The data are shown as the mean ± SD (n = 3–4), except for urine DPD in D20 at 0–12 h on day 29, and D40 at all time points, because the urine samples were not obtained. *Indicates p < 0.05 vs. vehicle for each administration frequency (a two-way ANOVA with a post-hoc Dunnett’s test). C, vehicle control; D20, daily 20 μg/kg administration; D40, daily 40 μg/kg administration; W140, weekly 140 μg/kg administration; W280, weekly 280 μg/kg administration.

To investigate the effects of the 4 TPTD regimens on bone metabolism, we monitored time-course of changes in the serum level of osteocalcin (OC) and the urinary level of deoxypyridinorine (DPD) after the administration of TPTD (Fig 2 and S3 Table).

The serum OC levels were monitored on days 1 and 22 after the initial administration of TPTD (Fig 2a). On day 1, all of the TPTD regimens reduced the serum OC levels to 24 h after administration (until the next daily dose in the D20 and D40 regimens). Under the weekly regimens (W140 and W280), the suppression gradually recovered and increased significantly to a higher level than the initial OC level on 3rd day after the administration of TPTD, but was maintained to day 7 (the next day of administration in the weekly dosing regimens). On day 22, the initial OC levels at 0 h in the daily regimens (D20 and D40) were higher than those in the weekly regimens (W140 and 280). The OC levels at 6 h in the daily regimens were transiently decreased to the level of the control C group, and then rapidly increased and recovered to the initial levels at 24 h; no such recovery was observed on day 1. In the weekly regimens (W140 and 280), the OC levels were also decreased at 6h; however, the recovery appeared slower than that observed in the daily group. The OC levels in the weekly regimens were highest on day 3 after administration, and then slowly decreased to the level of the control DV group on day 7. These findings in relation to the serum OC level indicated that sensitivity to the daily administration of TPTD differed on the different sampling days (1 and 22 days after the initial administration).

The urinary DPD levels were monitored on days 1 and 29 (Fig 2b). The DPD levels on day 1 were not significantly affected by any of the TPTD regimens. However, on day 29, the DPD levels at 0–12h in the D20, D40 and W280 groups were higher in comparison to the control C group. The DPD level at 12–24h in D40 maintained its higher level, but the DPD levels at 12–24h in the D20 and W280 groups were decreased to the control level. These urinary data indicated that urinary DPD levels become more sensitive to the administration of TPTD on day 29 after the initial administration in comparison to day 1.

### The obvious development of cortical porosity with daily regimens of TPTD observed by micro-Computed Tomography

**Fig 3:**
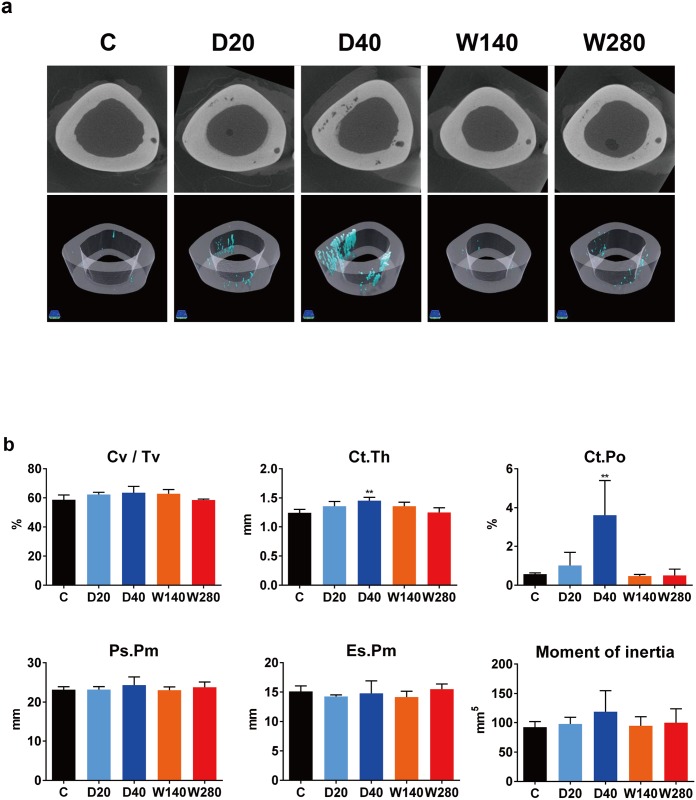
Micro- Computed Tomography (CT)-based analyses of the cortical bone of rabbit tibiae. (a) Three-dimensional reconstructed CT images of the bone structure of rabbit tibiae. The region of cortical porosity is highlighted in light blue. (b) The morphometric values of the cortical bone ratio (Cv /Tv, %), cortical bone thickness (Ct.Th, μm), cortical porosity (Ct.Po, %), perioteal perimeter (Ps.Pm, mm), endosteal perimeter (Es.Pm, mm) and the moment of inertia (mm^-5^) were measured and compared. The data are shown as the mean ± SD (n = 3–4). *Indicates p < 0.05 vs. vehicle control (ANOVA with post-hoc Dunnett’s test). C, vehicle control; D20, daily 20 μg/kg administration; D40, daily 40 μg/kg administration; W140, weekly 140 μg/kg administration; W280, weekly 280 μg/kg administration.

The effects of the 4 different TPTD regimens on the cortical structure were analyzed by micro-Computed Tomography (micro-CT) (Fig 3). Representative 2D grey scale and 3D-rendered images of tibial shafts obtained by micro-CT are shown in Fig 3a. The cortical voids in 3D images are highlighted by pseudocolor (light blue). The increased development of cortical voids along the longitudinal axis of tibial cortices was obviously observed in the daily regimens of TPTD administration, especially in the tibia obtained from D40.

The morphometrical parameters measured on micro-CT were analyzed and statistically compared (Fig 3b and S4 Table). The cortical bone volume / total tissue volume (Cv/Tv) was not significantly different from the control C in any of the regimens. The cortical bone thickness (Ct.Th) in the D40 (1.45±0.06 mm) group showed a significant increase in comparison to the C (1.25±0.06 mm). In the D40 group, the cortical porosity (3.62±1.78%) showed a significant and dramatic increase in comparison to the C group (0.57±0.06%). The same parameter was also increased in the D20 (1.02±0.67%); however, the difference did not reach statistical significance. The other parameters, such as the periosteal perimeter (Ps.Pm), the endosteal perimeter (Es.Pm) and moment of inertia did not show significant differences in comparison to the C control group in any of the regimens. These micro CT-based analyses indicated that cortical porosity was developed by the daily administration of TPTD, prior to the obvious increase in bone mass.

We also measured the bone mineral density (BMD) of tibiae, femora and lumber vertebrae (LV4)-LV6, and the mechanical strength of the tibiae (S1 and S2 Figs). The BMD and the mechanical strength of the bones tested were not significantly affected by 1-month treatment of TPTD in any group.

### Whole bright field and fluorescence views of bone sections provide spatial information on the development of the cortical void and the disorganized bone formation induced by the daily administration regimens

**Fig 4:**
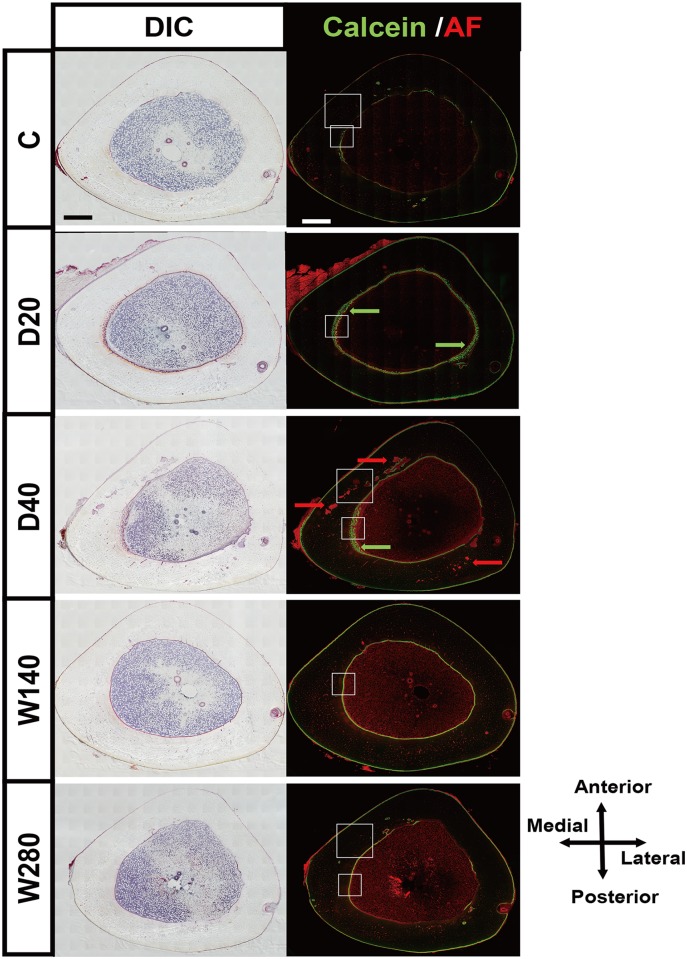
Bright and fluorescence images of the transverse sections of rabbit tibiae. Comprehensive tiling views of the bright field and fluorescence views were obtained from representative specimens in the vehicle control (DV), daily 20 μg/kg/day (D20), 40 μg/kg/day (D40), 140 μg/kg/week (W140), and 280 μg/kg/week (W280) groups (A, B, C, D, E, respectively). The green fluorescence signal from calcein labeling demarcates the sites of active bone formation, while the auto-fluorescence signal derived from the soft tissue provides morphological information. Bright field images were acquired with differential interference contrast (DIC). In the fluorescence view, calcein labelling- and soft-tissue-derived auto-fluorescence is shown in green and red, respectively. Wide-field fluorescence images were processed using the Deconvolution process. The white boxes and white dotted boxes indicate areas shown in Figs 5 and 6, respectively. Scale bars, 1000 μm.

To observe changes in the cortical microarchitecture in entire transvers sections of rabbit tibiae, we took advantage of tiling large high-resolution differential interference contrast (DIC) images and deconvolution fluorescence images (Fig 4). This imaging approach enabled us to manifest geometrical bone sites of active bone formation (labeled by calcein in green) and cortical porosity (demarcated by red auto-fluorescence in cortical bone). Clear single- or double-labelling using calcein was observed on the periosteal and endosteal surface of bone sections with each of the regimens, indicating the bone formation of cortices. It was noteworthy that jagged calcein labeling was observed on the endosteal surface of specimens obtained from daily regimens (D20 and D40) (indicated by green arrows in Fig 4). Furthermore, the obvious development of cortical porosity was observed in all of the specimens of the D40 regimen group and some of the specimens of the D20 group. Interestingly, the cortical porosity was predominantly seen in the anteromedial and posterolateral sites (indicated by red arrows in Fig 4).

**Fig 5:**
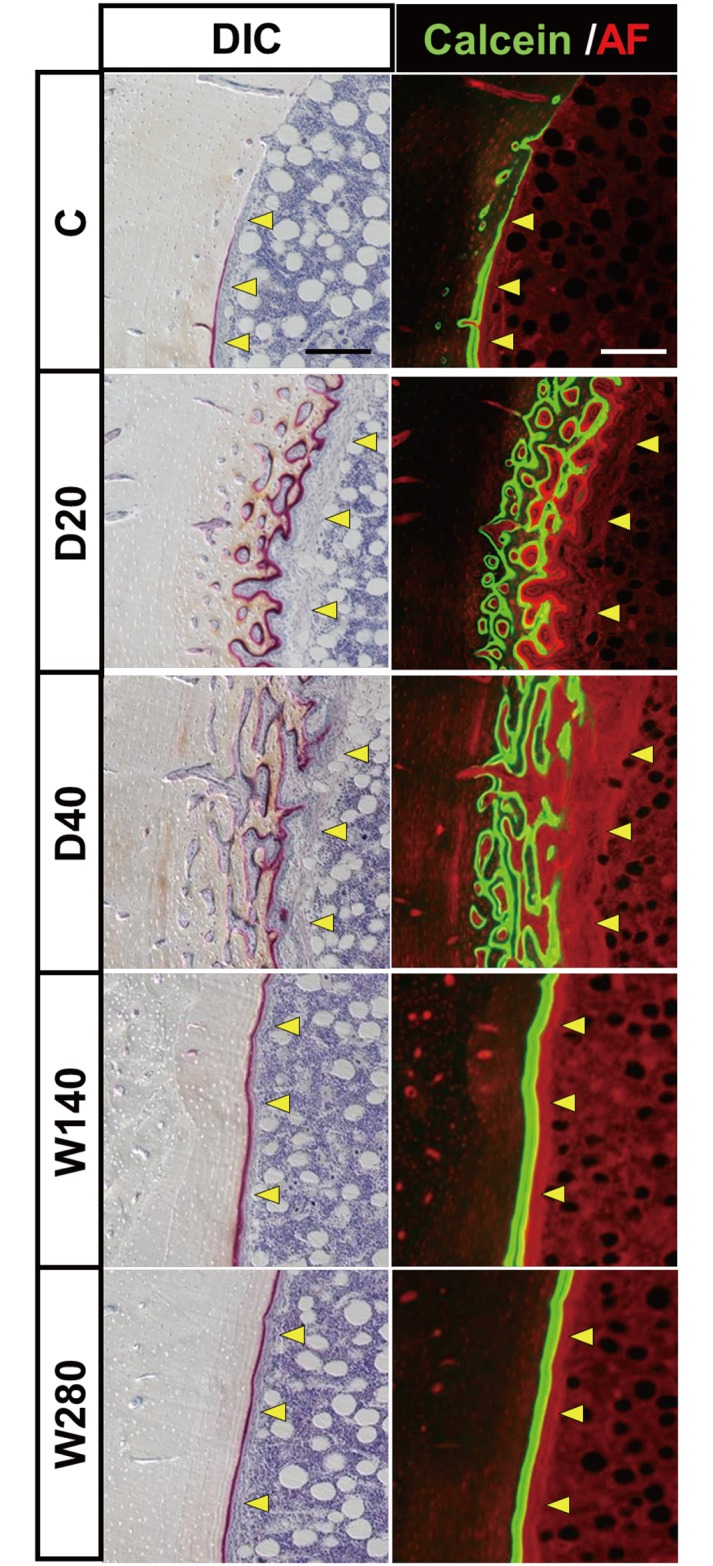
Magnified images of the endosteum region of the tibiae shown in Fig 4. Zoom-in views of the medial endosteum region from large views (the white boxed area in each panel) shown in Fig 4. The yellow arrowheads indicate the fibroblastic cell-enriched tissue layer covering the bone surface. These tissue layers in D20 and D40 exhibit the typical appearance of bone marrow fibrosis. Scale bars, 200 μm.

The enlargement of the medial portion of the endosteal surface of the large images shown in Fig 4 (see Fig 5) allowed jagged labelling to be observed in the D20 and D40 specimens, which showed multiple and irregular labelling on the bone surface, indicating disorganized ossification; in contrast, the corresponding bone surface in the W140 and W280 specimens showed linear double-labelling, indicating spatially harmonized ossification toward the bone marrow. It was also noted that multiple labeled areas in the D20 and D40 specimens were surrounded by thick fibroblastic tissue, indicating the formation of marrow fibrosis (indicated by yellow arrowheads). In contrast, double-labelling sites in the W140 and W280 specimens were covered by a thin fibroblastic tissue layer of typical endosteum in active bone formation (indicated by yellow arrowheads), which was also observed in the control C specimen. Furthermore, in the D20 and D40 specimens, a substantial portion of the mineralized area adjacent to the calcein-labeled area on DIC images exhibited red autofluorescence, indicating a poorly mineralized bone matrix. The well mineralized cortical bone matrix did not emit a fluorescence signal. These microscopic findings demonstrated that the daily administration of TPTD was associated with the development of bone marrow fibrosis and poorly mineralized and disorganized bone formation.

**Fig 6:**
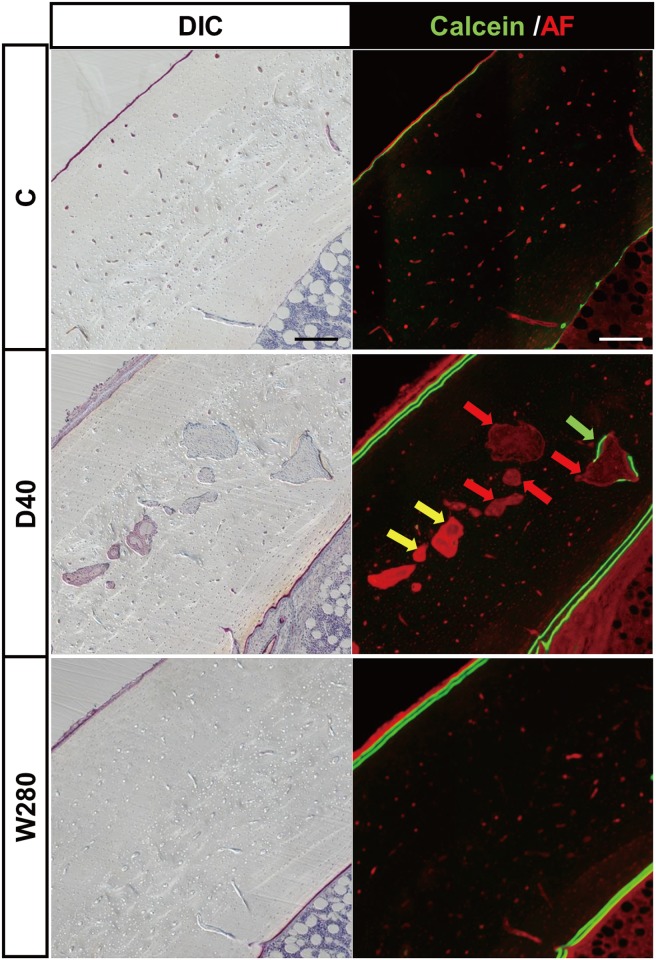
Magnified images of the cortical region of the tibiae shown in Fig 4. Zoom-in views of the anteromedial region of cortical bone from large views of the C, D40 and W280 groups (the dotted white boxed area in each panel) shown in Fig 4. The red arrows indicate the porotic cavities. The yellow and green arrows indicate the blood vessel and the calcein-labeled region in the porotic cavity, respectively. Scale bars, 200 μm.

Fig 6 shows enlarged views of the anteromedial cortical portions of specimens obtained from the C, D40 and W280 specimens shown in Fig 4. In the D40 histological sections, sites of cortical porosity were clearly demarcated by obviously large, red auto-fluorescence signals (indicated by red arrows in Fig 6), while the quiescent Haversian canals emitted small red signals that were observed in almost all of the intact cortical bone in the C and D280 specimens. DIC imaging demonstrated that the porous sites were largely filled with fibroblastic cells, some of which also blood vessels that were composed of endothelial cells (yellow arrow in Fig 6). Few of the porous sites showed calcein labelling (indicated by green arrow in Fig 6). These observations demonstrated that the daily administration of TPTD induced cortical porosity by enhancing the cortical turnover in the Haversian canals.

### The histomorphometrical analyses to investigate the development of the cortical void and disorganized bone formation induced by daily regimens of TPTD

**Fig 7:**
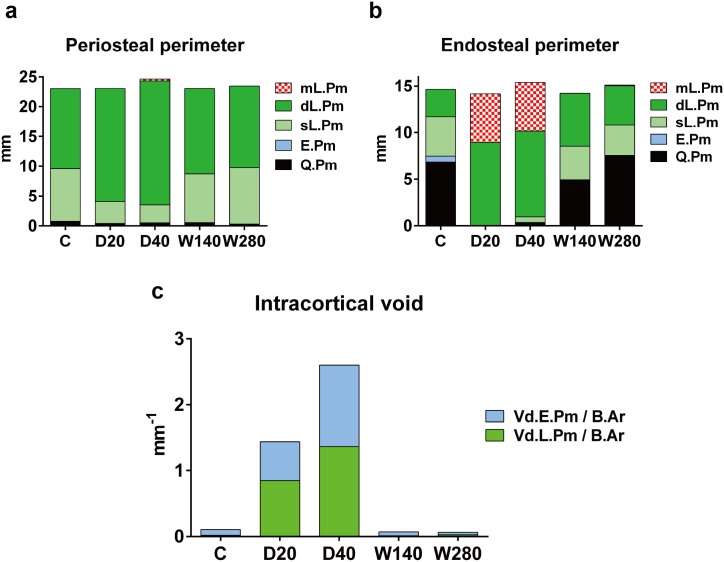
The histomorphometric analysis of the cortical bone parameters. The periosteal perimeter, endosteal perimeter and intracortical void were evaluated and compared. mL.Pm, multiple labeled perimeter; dL.Pm, double labeled perimeter; sL.Pm, single labeled perimeter; E.Pm, eroded perimeter; Q.Pm, quiescent perimeter. The data are shown as the mean (n = 3–4).

We carried out histomorphometrical analyses to observe the effects of the four regimens on the bone metabolism and microarchitecture (Fig 7 and S5 Table).

In the periosteal perimeter analysis (Fig 7a), the double-labeled surface (dL.Pm) was increased in the daily regimens (D20, D40) (18.99±3.83 and 20.81±2.64 mm, respectively) in comparison to (13.42±3.09 mm) the control C group, whereas the single-labeled surface (sL.Pm) in the daily regimens (3.64±4.97 and 3.02±2.91 mm, respectively) was decreased in comparison to the (8.82±2.75 mm) control C group. These parameters in the weekly groups (W140, W 280) (dL.Pm; 14.31±12.44 and 13.72±5.70 mm, sL.Pm; 8.16±12.30 and 9.42±5.02 mm, respectively) were comparable to those (dL.Pm; 13.42±3.09 mm, sL.Pm; 8.82±2.75 mm) in the control C group. These data indicated that the daily, but not weekly, administration of TPTD in our regimen settings stimulated the periosteal bone formation of the tibial cortices.

In the endosteal perimeter analysis (Fig 7b), approximately 50% of the total perimeter in the control C group was a quiescent perimeter (Q.Pm) (6.84±4.11 mm). However, in the daily regimens (D20, D40), the same parameters (0±0 and 0.29±0.50 mm, respectively) were almost lost, while the dL.Pm and multiple-labeling perimeter (mL.Pm) (dL.Pm; 8.94±5.71 and 9.19±1.00 mm, mL.Pm; 5.22±5.87 and 5.24±2.82 mm, respectively) were dramatically increased in comparison to the C (dL.Pm; 2.90±4.20 mm, mL.Pm; 0±0 mm), indicating that active but disorganized bone formation was stimulated in the intra-cortical bone area by the daily administrations of TPTD. In the weekly groups the dL.Pm (W140, 5.67±6.69; W280, 4.21±2.76 mm, respectively) was dramatically increased in comparison to the C group (2.90±4.20 mm), indicating that the weekly TPTD regimens promoted active and well-organized endosteal bone formation.

The area of the intracortical void in the daily regimens (D20, D40) were dramatically increased in a dose-dependent manner in comparison to the control C group (Fig 6c). The same parameters in the weekly regimens (W140, 280) were comparable to those in the C group. These data indicated that the daily, but not weekly, administrations of TPTD induced the development of cortical porosity. In the daily regimen groups (D20 and D40), the void eroded perimeter / bone area (Vd.E.Pm / B.Ar) (0.59±0.78 and 1.23±0.27 mm^-1^, respectively) and void labeled perimeter / bone area (Vd.L.Pm / B.Ar) (0.85±0.12 and 1.36±0.86 mm^-1^, respectively) were both highly increased in comparison to the control C group (Vd.E.Pm / B.Ar; 0.09 ± 0.06 mm^-1^, Vd.L.Pm / B.Ar; 0.02 ± 0.02 mm^-1^) and the weekly regimens (W140 and W280) (Vd.E.Pm / B.Ar; 0.06 ± 0.10 mm^-1^, Vd.L.Pm / B.Ar; 0.01 ± 0.02 mm^-1^, and Vd.E.Pm / B.Ar; 0.04 ± 0.03 mm^-1^, Vd.L.Pm / B.Ar; 0.03 ± 0.03 mm^-1^, respectively), suggesting that the daily administrations of TPTD induced activated cortical bone turnover.

### Naïve woven bone formation by marrow fibrosis observed by Three-Dimensional Second Harmonic Generation (3D-SHG) imaging

**Fig 8:**
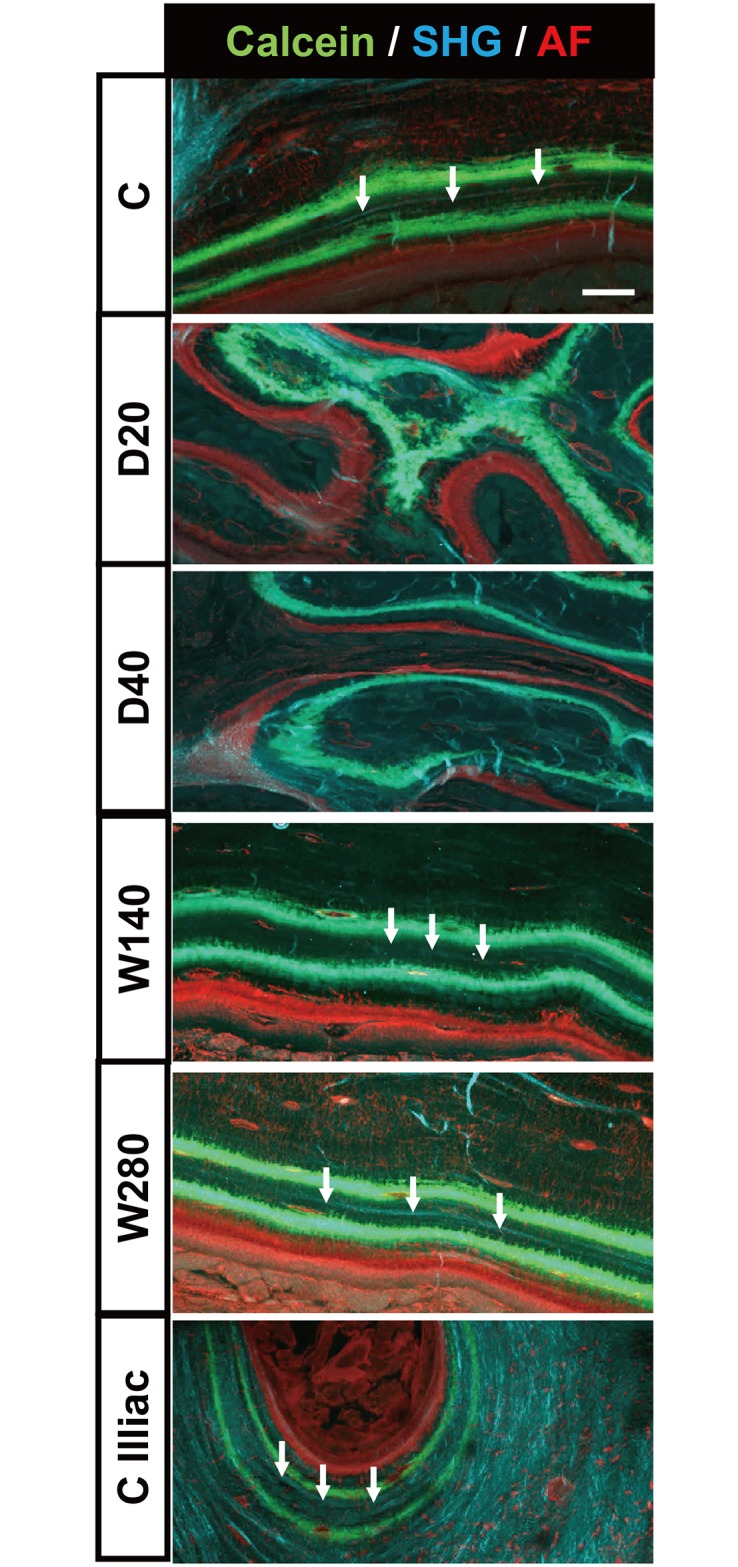
Three Dimensional Second Harmonic generation (3D-SHG) imaging detecting bone collagen pattern. SHG imaging of representative bone sections in the C, D20, D40, W140, and W280 groups and the trabecular bone of the iliac crest from the vehicle control (C iliac) group were acquired to detect bone collagen by focusing on the endosteal region. Three-dimensional projection images of 20 optical slices are shown. Bone collagen-derived SHG signaling, calcein labelling and soft tissue-derived auto-fluorescence are shown in blue, green and red, respectively. The white arrows indicate well-extended collagen fibers. Scale bars, 200 μm.

We next took advantage of three-dimensional second harmonic generation (3D-SHG) imaging using multiphoton microscopy to observe the pattern of endosteal bone collagen induced by the four TPTD regimens (Fig 8). In the C, W140 and W280 groups, linearly aligned collagen fibers were visualized (indicated by white arrows) between two stripes of calcein labeling, indicating well-organized lamellar bone formation. In contrast, in the daily groups (D20, D40), amorphously short fibers were observed in regions that were randomly labeled by calcein. These bone patterns were obviously different from the bone collagen patterns in the trabeculae of the iliac crest of the control C group, in which curved lamellar collagen patterns were observed (indicated by white arrows). These findings suggested that newly formed bone with multiple calcein labeling induced by the daily administration of TPTD was fibrous naïve bone rather than trabecular bone.

## Discussion

This study was designed to determine whether different frequencies of TPTD administration had different effects on the cortical bone microarchitecture such as the cortical porosity and bone marrow fibrosis. We aimed to observe the short-term effects (1 month for total administration period) on bone structure and metabolism prior to the significant increase in BMD and mechanical properties that can be expected after several months TPTD treatment. Morphometric analyses by micro-CT and histomorphometry demonstrated that the daily administration of TPTD (20 and 40 μg/kg/day; D20 and D40, respectively) was associated with the development of obvious cortical porosity and endosteal bone marrow fibrosis, which appeared to be dose-dependent. Their equivalent regimens in total weekly dosages (140 and 280 μg/kg/week, W140 and W280, respectively) did not lead these deleterious effects in the microarchitecture of the cortical bone.

We also monitored the pharmacokinetics of the plasma TPTD levels after the administration of TPTD. The dosages of our weekly regimens of TPTD (W140 and W280) were seven times higher than their equivalent daily regimens (D20 and D40). Accordingly, the maximum plasma concentrations (Cmax) and the area under the curve (AUC) of the weekly regimens were much higher in comparison to their equivalent daily regimens. However, the plasma TPTD levels appeared to return to the basal levels within 3 h of administration in all of the dosage regimens. Frolik et al., investigated several dosage regimens in rat models and demonstrated that the duration for which the serum level of human PTH (1–34) was elevated was a primary determinant of the anabolic and catabolic effects on the bone mass [32]. In their study, once-daily PTH or 6 PTH administrations/day within 1 h (total daily dosage; 80 μg/kg) increased the proximal tibia bone mass to an equivalent level. However, 6 PTH administrations/day over 6 h (total daily dosage; 80 μg/kg) and 3 PTH administrations/day over 8 h (total daily dosage; 240 μg/kg) decreased the tibia bone mass. They also suggested that the magnitude of the Cmax and AUC values of the serum PTH was not correlated with the anabolic or catabolic bone outcome, thus these were secondary determinants. Our analyses of the pharmacokinetics of TPTD and the histomorphometry of increases in dL.Pm suggested that, in all of our TPTD dosage regimens in rabbits, the duration for which the plasma levels of TPTD were elevated were short enough for the anabolic bone outcome to occur, although this short-term administration was too short for significant increases in bone mass to be observed. Thus, the concept presented by Frolik et al. using rats appeared to fit well with our rabbit study. In addition, our study also suggested that the development of cortical porosity and marrow fibrosis was more affected by the frequency of administration (daily or weekly) than the concentration of TPTD that was administered. Daily repetitive stimulation of the bone anabolic effect by TPTD can cause deleterious changes in the cortical microarchitecture.

We monitored the serum osteocalcin (OC) level after the administration of TPTD on days 1 and 22. An obvious distinction was observed between the daily and weekly dosage regimens with regard to the regulation of the serum OC level on day 22. The response of the serum OC level to the daily administration of TPTD on day 22 become more dramatic than that on day 1, suggesting that the daily administration of TPTD for 3 weeks might have had significantly increased the population of responder cells, possibly, osteoblast lineage cells, in comparison to the weekly administration of TPTD. The response of the urinary deoxypyridinorine (DPD) level to the administration of TPTD also become more sensitive on day 29 in comparison to day 1. This was the most significant in the D40 regimen, while the DPD level in weekly regimens were not significantly affected (W140) or returned to the baseline level soon after a transient increase (W280). The marked increase of the DPD level in the once-daily regimens suggests a significant increase in the population of osteoclastic cells. It is possible that these changes in the bone metabolism markers were associated with the development of cortical porosity and marrow fibrosis, which will be discussed in the following sections. The similar differences in the effect on bone metabolic markers between these administration frequencies were also shown in the previous clinical studies [3, 6, 8, 9].

The increase in the cortical porosity following the once-daily administration of TPTD has been well documented by previous reports using animal models and clinical studies [7, 20–23, 33]. However, it remained to be elucidated whether the lower frequency administration of TPTD (such as once weekly) has similar effects on the microstructure of the cortical bone. In our study, as described above, the once-daily administration of TPTD led to a significant increase in the cortical voids in the rabbit tibia, while the once-weekly administration did not cause this morphological change. Our observations by micro-CT and from the histological examination of bone sections indicated that the cortical porosity induced by the once-daily administration of TPTD was caused by accelerated bone resorption in the Haversian canals. Our dosing D40 regimen was associated with the most significant increases in cortical porosity and the urinary DPD level. This association suggests that the significant induction of DPD by daily regimens contributes to the increase in the population of osteoclast lineage cells in cortical void. Interestingly, the cortical voids were mostly seen in anteromedial and posterolateral area of tibia. This site-specific, or non-homogenous development of cortical porosity was also previously described in the rabbit femur [23]. However, the determinants of this phenomenon remain to be investigated. Local distinctions in mechanical loading and regional structural differences such as the distribution of blood vessels may be involved in the site-specific distribution of cortical porosity.

Our daily regimens of TPTD also caused a unique morphological change in the endosteal surfaces, forming a cancellous bone-like structure that was covered by a thick layer of fibroblast-like cells, a typical structure of bone marrow fibrosis. These surfaces showed intense multiple calcein-labeling, some of which also had eroded surfaces, especially in the sites that were connected with the intra-cortical voids, suggesting a strong enhancement of bone turnover in the regions. These cancellous bone-like regions had multiple and irregular calcein labelling on most of their surfaces both near to the original endosteal surface and within the deeper region near the marrow space. The bone formation in this region was quite irregular, since multiple points of ossification with a poorly mineralized matrix were observed. Furthermore, the bone collagen pattern observed by SHG imaging demonstrated that the spatial collagen patterns in this region were quite amorphous, which was in clear contrast to the linearly elongated collagen fibers along the lamellar structures of the cortical bone in the tibia and trabecular bone in the iliac crest of specimens obtained from the control C group. These findings indicated that the cancellous bone-like structure formed by marrow fibrosis is poorly developed woven bone. In contrast, our weekly regimens enhanced the bone formation in the endosteal surface, with an increase in the double calcein labelling perimeter (dL.Pm). This labelling was mostly continuous, and was parallel to the lamellar structure of previously formed cortical bone. Furthermore, SHG imaging demonstrated linearly extended bone collagen fibers along the calcein labelling and lamellar structure. These findings indicated that synchronized and entrained bone formation was induced by the weekly administration of TPTD from the bone surface toward the internal marrow space.

Cortical porosity and marrow fibrosis have also been reported in the bone tissue of hyperparathyroidism patients [26] and PPR*Tg mice, which constitutively express active PTH/PTHrP receptors [31]. The bone marrow fibrosis of PPR*Tg mice is reported to be attenuated by the administration of OPG which suppresses osteoclastogenesis via the competitive inhibition of receptor activator of nuclear factor kappa-B ligand (RANKL) binding to its receptor, RANK. Thus, the development of marrow fibrosis and cortical porosity in this transgenic mouse is related to the presence of osteoclasts that may secrete factors that facilitate the proliferation of fibroblastic cells. Another study in a rat model reported that the continuous infusion of TPTD causes bone marrow fibrosis [30]. This marrow fibrosis appeared after as little as 3 days and peaked within 1 week after the initial dosing of TPTD, while increased osteoclastogenesis was observed from 5 days and peaked on 2 weeks after the initial dose. Thus, the bone marrow fibrosis caused by TPTD treatment may not be associated with increased osteoclastogenesis. This study also demonstrated that the fibroblastic cells in bone marrow fibrosis contained cells that express osteoblast markers such as osteocalcin, osteonectin, and cbfa1, and that these cells eventually contributed to the formation of new bone. Thus, the fibroblastic cells, which are also described as fibroblastoid cells, in bone marrow fibrosis are of the osteoblast lineage. These findings in bone marrow fibrosis are consistent with our observation, especially in the daily regimens of TPTD. The responses of the serum OC level on day 22, and the urinary DPD level on day 29 to the daily administration of TPTD were more sensitive than those to daily regimens or any of the regimens on day 1. These phenomena may be related to the increased populations of osteoblasts in the tissue of bone marrow fibrosis that developed after the daily administration of TPTD. This tissue also contains osteoclasts, since we often observed an eroded surface in multiple calcein-labeled areas. The active coupling of bone resorption and bone formation may contribute to rapid but naïve bone formation.

Our weekly TPTD regimens were associated with the slight thickening of the endosteum, which contains several layers of fibroblastic cells. The E.Pm indicated that the eroded surface of this region was smaller. As described above, calcein labelling in this region was continuous and parallel to the inner bone surface. These findings suggest that the population of fibroblastic cells induced by the daily regimens was functionally different from the tissue of marrow fibrosis that developed from the weekly regimens. It is possible that bone formation in this region is not fully coupled with bone resorption, but that it proceeds through the activation of quiescent osteoblastic lining cells on the bone surface. This type of bone formation has been reported as mini-modeling in trabecular bone formation [34–36]. Without coupling with bone resorption, the rate of bone formation would be slow; this would concomitantly make synchronized and stable bone formation achievable. To support our interpretation, Yamamoto et al., recently reported that in a young adult male mouse model, the low-frequency administration of TPTD led to the formation of thicker trabecular bone through bone mini-modeling and remodeling, while the high-frequency administration of TPTD led to a rapid increase in bone formation by enhanced bone remodeling [12].

Because the period of this study was as short as 1 month, the long-term consequences of the increased cortical porosity, bone marrow fibrosis, and the naïve bone formation on the endosteal surface caused by daily TPTD treatment remain to be elucidated in future studies. The significance of the development of cortical porosity and bone marrow fibrosis requires further long-term studies in animal models and large clinical cohort studies. Our recent study using ovariectomized rats demonstrated that the cortical porosity was markedly developed by an increased administration frequency even with a lower concentration of total TPTD administration, although the highest concentration also induced cortical porosity [37]. Several studies have reported that the once-daily administration of TPTD induces trabecular tunneling in the iliac crest, femoral neck and vertebral sections obtained from monkey models of osteoporosis [33, 38, 39], but whether the same phenomenon is induced by once-weekly TPTD treatment has not been reported. Thus, a study using another species (such as monkeys) will be necessary to examine the effects of the frequency of TPTD administration on the trabecular structure.

In fact, it is controversial whether the PTH-induced increase in cortical porosity and marrow fibrosis actually impact the risk of bone fractures. It has been reported that, in terms of bone strength, the increase in cortical porosity caused by the daily administration of PTH is more than compensated by the increased formation of periosteal and endosteal bone in animal studies [20, 22, 23]. On the other hands, the cortical voids that were induced by the once-daily administration of TPTD have been reported to be increased chronologically and overall BMD in distal radius concomitantly continued to decrease until 18 months after the initiation of the once-daily treatment in clinical studies [7]. In addition, another clinical study showed that finite element estimated strength of radius and tibia decreased with the once-daily hPTH_1-84_ treatment on post-menopausal osteoporotic women [7, 40]. Cortical porosity in long bones was reported to increase with age [41] or with osteoporosis [42], and to have an influence on bone mechanical properties [18, 43]. Therefore, our current finding suggests that the frequency of TPTD treatment affecting cortical morphology of long bones have a potential impact on osteoporotic patients who already have porous cortices in their long bones, which emphasis the relevance to define the optimal frequency of PTH administration including the combinatorial daily and weekly applications for each clinical case [9].

Conclusively, the once-daily administration of TPTD for a short period of time (1 month) induced a marked increase in the cortical porosity, and marrow fibrosis-derived naïve bone formation on the endosteal surface, which were not observed following the once-weekly administration of equivalent total weekly doses. The marked augmentations in the serum OC and urinary DPD levels that were later observed after the daily administration of TPTD were possibly associated with the increased numbers of osteoblasts and osteoclasts in the porotic sites and the tissue of bone marrow fibrosis. Further long-term studies in appropriate animal models and clinical studies will be required to improve the treatment of osteoporosis in patients with a high risk of fracture in whom the porosity of the cortical bone may be increased.

## Supporting information

S1 Fig BMD of tibiae, femora, and lumbar vertebra. (TIF)

S2 Fig Mechanical properties of tibiae. (TIF)

S1 Table Plasma concentration of TPTD. (DOCX)

S2 Table Serum concentration of BUN and creatinine. (DOCX)

S3 Table Serum and urine concentration of bone metabolic markers. (DOCX)

S4 Table A micro-CT analysis of the cortical bone of the rabbit tibiae. (DOCX)

S5 Table Bone histomorphometry. (DOCX)
