# Supplementary figures and images for: Acute development of cortical porosity and endosteal naïve bone formation from the daily but not weekly short-term administration of PTH in rabbit
Source: PLoS One. 2017 Apr 10;12(4):e0175329. doi: 10.1371/journal.pone.0175329 (PMC5386260; doi:10.1371/journal.pone.0175329)

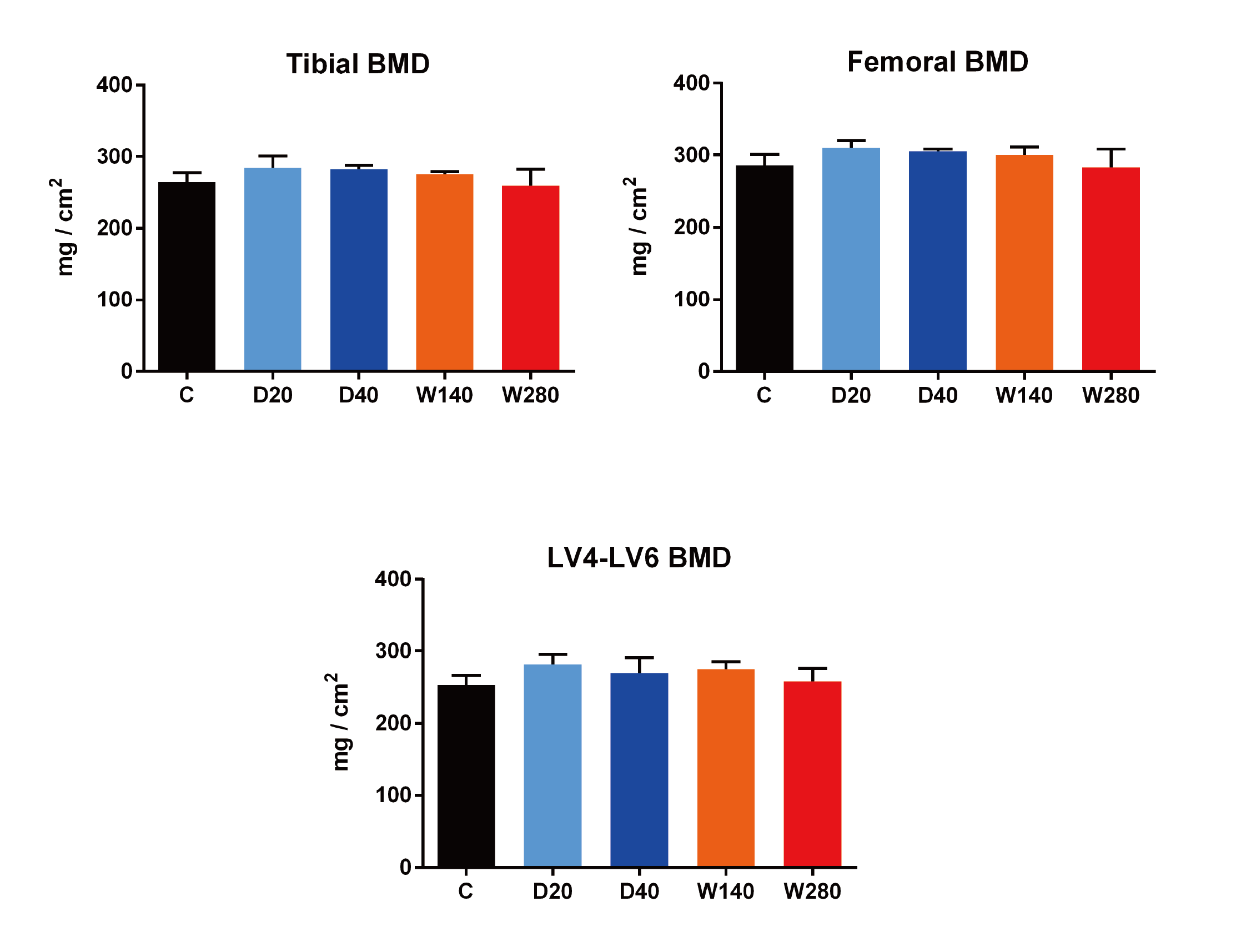

Supplement: S1 Fig — (TIF) [file pone.0175329.s001.tif]

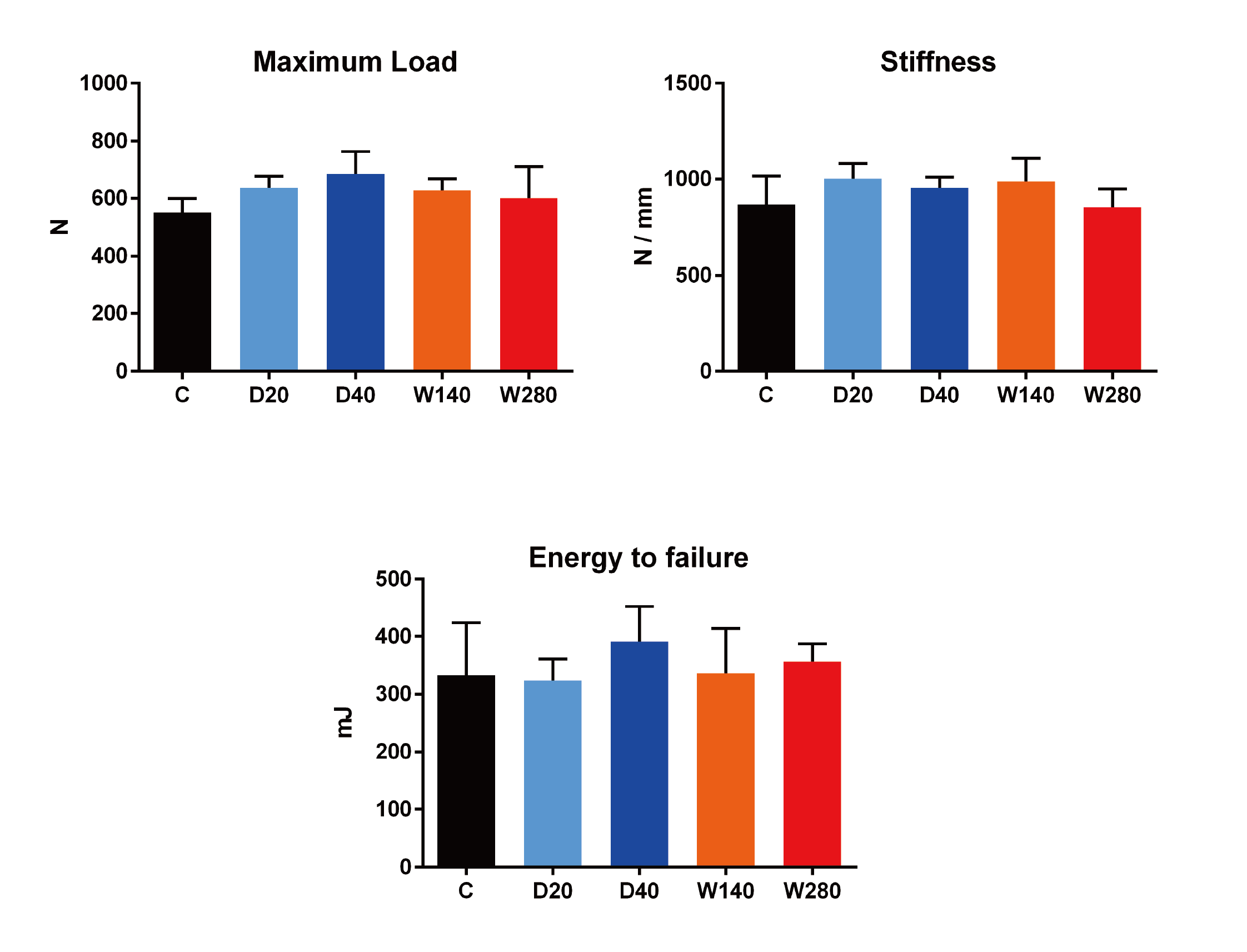

Supplement: S2 Fig — (TIF) [file pone.0175329.s002.tif]
