# Supplementary material for: Acute development of cortical porosity and endosteal naïve bone formation from the daily but not weekly short-term administration of PTH in rabbit
Source: PLoS One. 2017 Apr 10;12(4):e0175329. doi: 10.1371/journal.pone.0175329 (PMC5386260; doi:10.1371/journal.pone.0175329)
Supplement: S1 Table — (DOCX) [file pone.0175329.s003.docx]

**S1 Table. Plasma concentration of TPTD（see Figure 1b）**

**Mean ± SD**

**Day 1**

| **Time after administration** | **Units** | **D20** | | | **D40** | | | **W140** | | | **W280** | | |
| --- | --- | --- | --- | --- | --- | --- | --- | --- | --- | --- | --- | --- | --- |
| **Pre-**  **administration** | **ng/mL** | **N.D.** | **±** | **N.C.** | **N.D.** | **±** | **N.C.** | **N.D.** | **±** | **N.C.** | **N.D.** | **±** | **N.C.** |
| **5 min** |  | **7.14** | **±** | **2.36** | **9.83** | **±** | **5.64** | **37.99** | **±** | **20.04** | **60.62** | **±** | **24.23** |
| **15 min** |  | **14.87** | **±** | **3.33** | **23.62** | **±** | **13.22** | **85.36** | **±** | **26.72** | **177.00** | **±** | **66.73** |
| **30 min** |  | **19.78** | **±** | **5.67** | **34.30** | **±** | **8.19** | **131.96** | **±** | **30.23** | **294.58** | **±** | **79.89** |
| **1 hour** |  | **11.77** | **±** | **1.53** | **29.93** | **±** | **6.90** | **120.91** | **±** | **18.13** | **254.76** | **±** | **49.81** |
| **2 hour** |  | **1.77** | **±** | **0.91** | **10.58** | **±** | **3.28** | **50.39** | **±** | **8.19** | **87.37** | **±** | **57.48** |
| **24 hour** |  | **ND** | **±** | **NC** | **ND** | **±** | **NC** | **ND** | **±** | **NC** | **ND** | **±** | **NC** |

**Day 22**

| **Time after administration** | **Units** | **D20** | | | **D40** | | | **W140** | | | **W280** | | |
| --- | --- | --- | --- | --- | --- | --- | --- | --- | --- | --- | --- | --- | --- |
| **Pre-**  **administration** | **ng/mL** | **0.13** | **±** | **0.02** | **N.D.** | **±** | **N.C.** | **N.D.** | **±** | **N.C.** | **N.D.** | **±** | **N.C.** |
| **5 min** |  | **7.06** | **±** | **2.31** | **10.30** | **±** | **4.71** | **33.48** | **±** | **13.03** | **67.73** | **±** | **23.40** |
| **15 min** |  | **17.01** | **±** | **4.49** | **32.66** | **±** | **14.08** | **124.04** | **±** | **38.66** | **231.65** | **±** | **75.72** |
| **30 min** |  | **24.55** | **±** | **6.35** | **54.05** | **±** | **22.70** | **201.63** | **±** | **57.56** | **356.14** | **±** | **98.85** |
| **1 hour** |  | **20.47** | **±** | **6.21** | **49.17** | **±** | **21.58** | **147.11** | **±** | **22.78** | **279.91** | **±** | **62.38** |
| **2 hour** |  | **3.80** | **±** | **1.94** | **14.04** | **±** | **5.01** | **37.77** | **±** | **13.19** | **62.41** | **±** | **31.21** |
| **24 hour** |  | **0.13** | **±** | **0.01** | **ND** | **±** | **NC** | **ND** | **±** | **NC** | **ND** | **±** | **NC** |

**ND: Not detected, NC: Not calculated**
