# Supplementary material for: Acute development of cortical porosity and endosteal naïve bone formation from the daily but not weekly short-term administration of PTH in rabbit
Source: PLoS One. 2017 Apr 10;12(4):e0175329. doi: 10.1371/journal.pone.0175329 (PMC5386260; doi:10.1371/journal.pone.0175329)
Supplement: S2 Table — (DOCX) [file pone.0175329.s004.docx]

**S2 Table. Serum concentration of BUN and creatinine.**

**Serum concentration of blood urea nitrogen (BUN)**

**Mean ± SD**

**Day 1**

| **Time** | **Units** | **DV** | | | **D20** | | | **D40** | | | **W140** | | | **W280** | | |
| --- | --- | --- | --- | --- | --- | --- | --- | --- | --- | --- | --- | --- | --- | --- | --- | --- |
| **0** | **mg/dL** | **22.10** | **±** | **1.74** | **21.60** | **±** | **1.87** | **22.17** | **±** | **1.75** | **20.03** | **±** | **1.63** | **22.38** | **±** | **0.67** |
| **6 hour** |  | **26.28** | **±** | **0.57** | **23.40** | **±** | **3.36** | **26.40** | **±** | **1.73** | **27.83** | **±** | **1.50** | **27.55** | **±** | **2.03** |
| **1 day** |  | **21.35** | **±** | **3.10** | **19.67** | **±** | **2.12** | **20.20** | **±** | **1.31** | **19.80** | **±** | **1.15** | **22.95** | **±** | **1.11** |
| **3 day** |  | **22.88** | **±** | **2.22** |  | | |  | | | **21.53** | **±** | **0.40** | **22.90** | **±** | **1.42** |
| **7 day** |  | **22.78** | **±** | **1.51** |  |  |  |  |  |  | **19.97** | **±** | **1.88** | **21.80** | **±** | **3.01** |

**Day 22**

| **Time** | **Units** | **DV** | | | **D20** | | | **D40** | | | **W140** | | | **W280** | | |
| --- | --- | --- | --- | --- | --- | --- | --- | --- | --- | --- | --- | --- | --- | --- | --- | --- |
| **0** | **mg/dL** | **21.13** | **±** | **4.96** | **20.47** | **±** | **0.81** | **18.33** | **±** | **2.48** | **20.70** | **±** | **1.45** | **21.65** | **±** | **1.18** |
| **6 hour** |  | **25.25** | **±** | **5.50** | **24.20** | **±** | **1.23** | **20.87** | **±** | **3.67** | **25.50** | **±** | **3.42** | **28.20** | **±** | **2.39** |
| **1 day** |  | **21.65** | **±** | **4.62** | **21.30** | **±** | **1.05** | **18.67** | **±** | **3.06** | **19.60** | **±** | **1.80** | **21.23** | **±** | **0.85** |
| **3 day** |  | **22.63** | **±** | **2.90** |  | | |  | | | **20.13** | **±** | **1.63** | **21.20** | **±** | **2.16** |
| **7 day** |  | **23.20** | **±** | **2.50** |  |  |  |  |  |  | **21.60** | **±** | **2.93** | **22.30** | **±** | **1.96** |

**Serum concentration of creatinine**

**Mean ± SD**

**Day 1**

| **Time** | **Units** | **DV** | | | **D20** | | | **D40** | | | **W140** | | | **W280** | | |
| --- | --- | --- | --- | --- | --- | --- | --- | --- | --- | --- | --- | --- | --- | --- | --- | --- |
| **0** | **mg/dL** | **1.33** | **±** | **0.08** | **1.16** | **±** | **0.19** | **1.08** | **±** | **0.13** | **1.45** | **±** | **0.26** | **1.30** | **±** | **0.12** |
| **6 hour** |  | **1.12** | **±** | **0.06** | **1.18** | **±** | **0.06** | **1.07** | **±** | **0.04** | **1.15** | **±** | **0.19** | **1.12** | **±** | **0.15** |
| **1 day** |  | **1.13** | **±** | **0.04** | **1.14** | **±** | **0.09** | **1.05** | **±** | **0.09** | **1.26** | **±** | **0.17** | **1.13** | **±** | **0.12** |
| **3 day** |  | **1.19** | **±** | **0.09** |  | | |  | | | **1.27** | **±** | **0.16** | **1.11** | **±** | **0.14** |
| **7 day** |  | **1.30** | **±** | **0.15** |  |  |  |  |  |  | **1.36** | **±** | **0.17** | **1.21** | **±** | **0.17** |

**Day 22**

| **Time** | **Units** | **DV** | | | **D20** | | | **D40** | | | **W140** | | | **W280** | | |
| --- | --- | --- | --- | --- | --- | --- | --- | --- | --- | --- | --- | --- | --- | --- | --- | --- |
| **0** | **mg/dL** | **1.36** | **±** | **0.22** | **1.16** | **±** | **0.11** | **1.09** | **±** | **0.12** | **1.38** | **±** | **0.20** | **1.28** | **±** | **0.14** |
| **6 hour** |  | **1.08** | **±** | **0.10** | **1.12** | **±** | **0.09** | **0.97** | **±** | **0.04** | **1.07** | **±** | **0.15** | **1.18** | **±** | **0.17** |
| **1 day** |  | **1.35** | **±** | **0.28** | **1.21** | **±** | **0.07** | **1.06** | **±** | **0.14** | **1.46** | **±** | **0.30** | **1.23** | **±** | **0.18** |
| **3 day** |  | **1.35** | **±** | **0.18** |  | | |  | | | **1.30** | **±** | **0.20** | **1.21** | **±** | **0.15** |
| **7 day** |  | **1.20** | **±** | **0.18** |  |  |  |  |  |  | **1.15** | **±** | **0.14** | **1.11** | **±** | **0.17** |
