# Supplementary material for: Acute development of cortical porosity and endosteal naïve bone formation from the daily but not weekly short-term administration of PTH in rabbit
Source: PLoS One. 2017 Apr 10;12(4):e0175329. doi: 10.1371/journal.pone.0175329 (PMC5386260; doi:10.1371/journal.pone.0175329)
Supplement: S3 Table — (DOCX) [file pone.0175329.s005.docx]

**S3 Table. Serum and urine concentration of bone metabolic markers.**

**Serum concentration of osteocalcin (OC)（see Figure 2a）**

**Mean ± SD**

**Day 1**

| **Time** | **Units** | **DV** | | | **D20** | | | **D40** | | | **W140** | | | **W280** | | |
| --- | --- | --- | --- | --- | --- | --- | --- | --- | --- | --- | --- | --- | --- | --- | --- | --- |
| **0** | **%** | **100** | **±** | **0** | **100** | **±** | **0** | **100** | **±** | **0** | **100** | **±** | **0** | **100** | **±** | **0** |
| **6 hour** |  | **56.59** | **±** | **17.72** | **50.20** | **±** | **28.54** | **40.28** | **±** | **3.95** | **43.38** | **±** | **6.23** | **43.33** | **±** | **40.53** |
| **1 day** |  | **52.25** | **±** | **30.12** | **49.17** | **±** | **48.92** | **43.69** | **±** | **5.56** | **67.25** | **±** | **26.33** | **36.31** | **±** | **8.93** |
| **3 day** |  | **42.35** | **±** | **15.40** |  | | |  | | | **129.66** | **±** | **32.55** | **90.21** | **±** | **43.16** |
| **7 day** |  | **54.08** | **±** | **12.43** |  |  |  |  |  |  | **106.83** | **±** | **13.98** | **77.30** | **±** | **36.95** |

**Day22**

| **Time** | **Units** | **DV** | | | **D20** | | | **D40** | | | **W140** | | | **W280** | | |
| --- | --- | --- | --- | --- | --- | --- | --- | --- | --- | --- | --- | --- | --- | --- | --- | --- |
| **0** | **%** | **59.29** | **±** | **32.31** | **152.72** | **±** | **90.52** | **145.93** | **±** | **26.96** | **90.25** | **±** | **21.71** | **60.08** | **±** | **26.71** |
| **6 hour** |  | **45.20** | **±** | **17.19** | **82.17** | **±** | **39.32** | **64.45** | **±** | **11.83** | **37.57** | **±** | **10.10** | **25.20** | **±** | **10.68** |
| **1 day** |  | **40.53** | **±** | **15.02** | **168.69** | **±** | **102.38** | **146.74** | **±** | **15.23** | **58.12** | **±** | **3.01** | **27.01** | **±** | **11.89** |
| **3 day** |  | **51.03** | **±** | **18.26** |  | | |  | | | **109.02** | **±** | **15.02** | **64.09** | **±** | **29.08** |
| **7 day** |  | **47.16** | **±** | **22.55** |  |  |  |  |  |  | **76.25** | **±** | **11.65** | **45.53** | **±** | **20.47** |

**Urine concentration of deoxypyridinorine (DPD)（see Figure 2b）**

**Mean ± SD**

**Day1**

| **Time** | **Units** | **DV** | | | **D20** | | | **D40** | | | **W140** | | | **W280** | | |
| --- | --- | --- | --- | --- | --- | --- | --- | --- | --- | --- | --- | --- | --- | --- | --- | --- |
| **Day1**  **0-12h** | **%** | **90.72** | **±** | **11.51** | **91.47** | **±** | **14.40** | **111.65** | **±** | **8.71** | **89.43** | **±** | **1.68** | **108.70** | **±** | **9.30** |
| **Day1**  **12-24h** |  | **98.69** | **±** | **8.98** | **94.23** | **±** | **7.27** | **113.66** | **±** | **1.89** | **92.14** | **±** | **6.18** | **107.86** | **±** | **12.84** |

**Day29**

| **Time** | **Units** | **DV** | | | **D20** | | | **D40** | | | | **W140** | | | **W280** | | | |  |
| --- | --- | --- | --- | --- | --- | --- | --- | --- | --- | --- | --- | --- | --- | --- | --- | --- | --- | --- | --- |
| **Day29**  **0-12h** | **%** | **65.86** | **±** | **11.06** | **137.27** | | | **124.95** | | **±** | **34.51** | **80.74** | **±** | **12.49** | **113.32** | **±** | **16.06** | |  |
| **Day29**  **12-24h** |  | **72.23** | **±** | **11.64** | **86.98** | **±** | **19.61** | | **124.97** | **±** | **7.80** | **78.10** | **±** | **11.15** | **86.53** | **±** | | **17.50** | |
