# Supplementary material for: Acute development of cortical porosity and endosteal naïve bone formation from the daily but not weekly short-term administration of PTH in rabbit
Source: PLoS One. 2017 Apr 10;12(4):e0175329. doi: 10.1371/journal.pone.0175329 (PMC5386260; doi:10.1371/journal.pone.0175329)
Supplement: S4 Table — (DOCX) [file pone.0175329.s006.docx]

**S4 Table. A micro-CT analysis of the cortical bone of the rabbit tibiae (see Fig 3b).**

**Mean ± SD**

| **Parameters** | **Units** | **DV** | | | **D20** | | | **D40** | | | **W140** | | | **W280** | | |
| --- | --- | --- | --- | --- | --- | --- | --- | --- | --- | --- | --- | --- | --- | --- | --- | --- |
| **Cv/Tv** | **%** | **58.72** | **±** | **3.24** | **62.31** | **±** | **1.54** | **63.54** | **±** | **4.33** | **62.84** | **±** | **2.83** | **58.47** | **±** | **0.68** |
| **Ct.Th** | **mm** | **1.25** | **±** | **0.06** | **1.36** | **±** | **0.08** | **1.45** | **±** | **0.06** | **1.36** | **±** | **0.07** | **1.25** | **±** | **0.08** |
| **Cortical**  **porosity** | **%** | **0.57** | **±** | **0.06** | **1.02** | **±** | **0.67** | **3.62** | **±** | **1.78** | **0.47** | **±** | **0.08** | **0.50** | **±** | **0.33** |
| **Ps.Pm** | **mm** | **23.16** | **±** | **0.75** | **23.19** | **±** | **0.76** | **24.31** | **±** | **2.09** | **23.03** | **±** | **0.82** | **23.79** | **±** | **1.33** |
| **Es.Pm** | **mm** | **15.10** | **±** | **0.96** | **14.26** | **±** | **0.25** | **14.78** | **±** | **2.13** | **14.15** | **±** | **0.98** | **15.51** | **±** | **0.87** |
| **Moment of Inertia** | **mm^5^** | **92.17** | **±** | **9.50** | **97.82** | **±** | **11.31** | **118.66** | **±** | **36.14** | **94.73** | **±** | **15.35** | **99.84** | **±** | **23.78** |
