# Supplementary material for: Acute development of cortical porosity and endosteal naïve bone formation from the daily but not weekly short-term administration of PTH in rabbit
Source: PLoS One. 2017 Apr 10;12(4):e0175329. doi: 10.1371/journal.pone.0175329 (PMC5386260; doi:10.1371/journal.pone.0175329)
Supplement: S5 Table — (DOCX) [file pone.0175329.s007.docx]

**S5 Table. Bone histomorphometry (see Fig 7).**

**Mean ± SD**

**Periosteal perimeters**

| **Parameters** | **Units** | **DV** | | | **D20** | | | **D40** | | | **W140** | | | **W280** | | |
| --- | --- | --- | --- | --- | --- | --- | --- | --- | --- | --- | --- | --- | --- | --- | --- | --- |
| **mL.Pm** | **mm** | **0** | **±** | **0** | **0** | **±** | **0** | **0.30** | **±** | **0.52** | **0** | **±** | **0** | **0** | **±** | **0** |
| **dL.Pm** |  | **13.42** | **±** | **3.09** | **18.99** | **±** | **3.83** | **20.81** | **±** | **2.64** | **14.31** | **±** | **12.44** | **13.72** | **±** | **5.70** |
| **sL.Pm** |  | **8.82** | **±** | **2.75** | **3.64** | **±** | **4.97** | **3.02** | **±** | **2.91** | **8.16** | **±** | **12.30** | **9.42** | **±** | **5.02** |
| **E.Pm** |  | **0** | **±** | **0** | **0.14** | **±** | **0.24** | **0** | **±** | **0** | **0** | **±** | **0** | **0** | **±** | **0** |
| **Q.Pm** |  | **0.78** | **±** | **0.90** | **0.28** | **±** | **0.49** | **0.50** | **±** | **0.87** | **0.56** | **±** | **0.97** | **0.32** | **±** | **0.64** |

**Endosteal perimeters**

| **Parameters** | **Units** | **DV** | | | **D20** | | | **D40** | | | **W140** | | | **W280** | | |
| --- | --- | --- | --- | --- | --- | --- | --- | --- | --- | --- | --- | --- | --- | --- | --- | --- |
| **mL.Pm** | **mm** | **0** | **±** | **0** | **5.22** | **±** | **5.87** | **5.24** | **±** | **2.82** | **0** | **±** | **0** | **0.07** | **±** | **0.14** |
| **dL.Pm** |  | **2.90** | **±** | **4.20** | **8.94** | **±** | **5.71** | **9.19** | **±** | **1.00** | **5.67** | **±** | **6.69** | **4.21** | **±** | **2.76** |
| **sL.Pm** |  | **4.24** | **±** | **1.32** | **0** | **±** | **0** | **0.59** | **±** | **0.58** | **3.60** | **±** | **3.06** | **3.28** | **±** | **1.27** |
| **E.Pm** |  | **0.64** | **±** | **0.50** | **0** | **±** | **0** | **0.09** | **±** | **0.15** | **0.05** | **±** | **0.09** | **0** | **±** | **0** |
| **Q.Pm** |  | **6.84** | **±** | **4.11** | **0** | **±** | **0** | **0.29** | **±** | **0.50** | **4.89** | **±** | **4.94** | **7.55** | **±** | **2.29** |

**Cortical void**

| **Parameters** | **Units** | **DV** | | | **D20** | | | **D40** | | | **W140** | | | **W280** | | |
| --- | --- | --- | --- | --- | --- | --- | --- | --- | --- | --- | --- | --- | --- | --- | --- | --- |
| **E.Pm/B.Ar** | **mm^-1^** | **0.09** | **±** | **0.06** | **0.59** | **±** | **0.78** | **1.23** | **±** | **0.27** | **0.06** | **±** | **0.10** | **0.04** | **±** | **0.03** |
| **L.Pm/B.Ar** |  | **0.02** | **±** | **0.02** | **0.85** | **±** | **0.12** | **1.36** | **±** | **0.86** | **0.01** | **±** | **0.02** | **0.03** | **±** | **0.03** |
